# Evaluating Diagnostic Accuracy and Treatment Efficacy in Mental Health: A Comparative Analysis of Large Language Model Tools and Mental Health Professionals

**DOI:** 10.3390/ejihpe15010009

**Published:** 2025-01-18

**Authors:** Inbar Levkovich

**Affiliations:** Faculty of Education, Tel-Hai Academic College, Upper Galilee 2208, Israel; levkovinb@telhai.ac.il

**Keywords:** large language models, artificial intelligence, mental health, depression, suicide, schizophrenia, social phobia, PTSD

## Abstract

Large language models (LLMs) offer promising possibilities in mental health, yet their ability to assess disorders and recommend treatments remains underexplored. This quantitative cross-sectional study evaluated four LLMs (Gemini (Gemini 2.0 Flash Experimental), Claude (Claude 3.5 Sonnet), ChatGPT-3.5, and ChatGPT-4) using text vignettes representing conditions such as depression, suicidal ideation, early and chronic schizophrenia, social phobia, and PTSD. Each model’s diagnostic accuracy, treatment recommendations, and predicted outcomes were compared with norms established by mental health professionals. Findings indicated that for certain conditions, including depression and PTSD, models like ChatGPT-4 achieved higher diagnostic accuracy compared to human professionals. However, in more complex cases, such as early schizophrenia, LLM performance varied, with ChatGPT-4 achieving only 55% accuracy, while other LLMs and professionals performed better. LLMs tended to suggest a broader range of proactive treatments, whereas professionals recommended more targeted psychiatric consultations and specific medications. In terms of outcome predictions, professionals were generally more optimistic regarding full recovery, especially with treatment, while LLMs predicted lower full recovery rates and higher partial recovery rates, particularly in untreated cases. While LLMs recommend a broader treatment range, their conservative recovery predictions, particularly for complex conditions, highlight the need for professional oversight. LLMs provide valuable support in diagnostics and treatment planning but cannot replace professional discretion.

## 1. Introduction

The advent of large language models (LLMs) has heralded a new era of technological advancement with far-reaching implications across various fields, including psychiatry (Obradovich et al., 2024). These sophisticated artificial intelligence systems, trained on extensive textual data corpora, have exhibited significant capabilities in natural language processing, generation, and understanding (Luo et al., 2024). Given these advancements, this study aimed to empirically test how LLMs are applied in the critical area of mental health diagnostics and treatment recommendations.

Large language models (LLMs), such as ChatGPT-3.5 and ChatGPT-4, have been extensively trained on diverse textual datasets using advanced transformer architectures and self-supervised learning methods (Luo et al., 2024; Omar & Levkovich, 2024). These pretrained models excel in processing and generating human-like language, enabling their application in critical areas such as mental health diagnostics and treatment planning (Elyoseph et al., 2024; Obradovich et al., 2024). However, the proprietary nature of their training processes limits transparency, often presenting a “black box” characteristic that necessitates careful interpretation of their outputs in clinical applications (Levkovich et al., 2024a). Despite these limitations, research highlights their potential to perform comparably to mental health professionals in areas such as suicide risk assessment and treatment recommendations, underscoring their transformative capabilities (Elyoseph et al., 2024; Levkovich et al., 2024b).

The rapid development of LLM tools, such as ChatGPT-4, Claude, and their derivatives, offers unprecedented opportunities for advancing therapeutic interventions, optimizing data analysis, and facilitating personalized patient care (Demszky et al., 2023; Yang et al., 2023). Moreover, the ability of LLMs to integrate multimodal data, such as patient history and symptom descriptions, further enhances their diagnostic utility, highlighting their potential to address existing gaps in mental healthcare (Nazi & Peng, 2024).

Nevertheless, as AI-driven solutions gain prominence, these technologies also prompt critical ethical considerations, including concerns about data privacy and the potential diminishment of human expertise (Hadar-Shoval et al., 2024; Tortora, 2024). Additionally, although LLMs can enhance diagnostic accuracy and treatment options, they cannot replicate the nuanced understanding and empathetic engagement that human clinicians provide, making it essential to consider their role as supportive tools rather than replacements for mental health professionals (Haber et al., 2024).

Indeed, accurate diagnosis by mental health professionals is essential for delivering effective treatment and care as it directly influences patient well-being and treatment outcomes (Haber et al., 2024). Yet the diagnostic process is often complicated by such challenges as overlapping symptoms across disorders and the influence of cultural biases, which can lead to potential inaccuracies and subsequently affect the quality of patient care (Bradford et al., 2024). LLM assessments do not always align with those of human mental health clinicians, indicating the need for further refinement before LLMs can operate independently. In one study (Elyoseph & Levkovich, 2024), ChatGPT was used to analyze case vignettes with varying levels of perceived burdensomeness and thwarted belonging, both of which are key suicide risk factors. Although ChatGPT correctly identified the highest risk in vignettes with elevated levels of both factors, it generally predicted a lower suicide risk than human professionals (Elyoseph & Levkovich, 2023). In contrast, in another study related to depression, the diagnoses and recommendations made by ChatGPT were found to be more accurate than those of medical professionals (Levkovich & Elyoseph, 2023).

Beyond the ability of professionals to identify mental health issues, the treatment recommendations they provide constitute a critical step in the therapeutic process (Morgan et al., 2013; Elyoseph & Levkovich, 2024). Previous studies comparing professionals and language models have yielded conflicting results. In research related to depression, the diagnoses and recommendations in identifying depression and its determinants made by ChatGPT were found to be more accurate than those of medical professionals. One study (Elyoseph & Levkovich, 2024) evaluated the ability of mental health professionals to predict the prognosis of schizophrenia, both with and without treatment, including long-term positive and negative outcomes. The study then compared these professional evaluations with those of Google Bard, ChatGPT-3.5, ChatGPT-4, and Claude (Elyoseph & Levkovich, 2024). The findings revealed that while ChatGPT-3.5 produced more pessimistic estimates, the other language models were closely aligned with the professional evaluations (Elyoseph & Levkovich, 2024). A review examining how artificial intelligence tools were applied in managing anxiety and depression revealed that a wide range of tools, including chatbots, mobile applications, and LLMs, are effective in reducing symptoms (Pavlopoulos et al., 2024).

To date, only limited research has examined diagnosis and treatment of psychiatric conditions using LLM tools. Most studies have focused on specific mental disorders, such as depression and schizophrenia, and have primarily assessed the ability of various LLM tools to identify these conditions and evaluate treatment recommendations. Previous studies comparing professionals and language models have yielded conflicting results (Omar et al., 2024).

The current study offers a comprehensive examination of multiple disorders across a broad range of models. In doing so, it explores the intersection between LLM tools and mental health professionals, with particular focus on how these technologies have reshaped the landscape of mental healthcare, psychological assessment, and predicted outcomes. By analyzing the experiences and perspectives of psychology professionals as a reference group, we seek to provide a comprehensive understanding of the current state of LLM integration in mental health.

The research objectives were as follows:

1. To compare correct diagnosis rates across different LLM tools and mental health professionals.
2. To compare treatments across different LLM tools and mental health professionals.
3. To compare outcomes predicted by LLM tools and mental health professionals, both for those who received help and for those who did not.

## 2. Materials and Methods

### 2.1. Procedure

The study was conducted in May 2024 to investigate how four advanced LLMs were applied in mental health diagnostics: Gemini (Google), Claude (Anthropic), ChatGPT-3.5, and ChatGPT-4 (OpenAI). The LLMs were chosen based on their recent advancements and specific capabilities in processing natural language inputs related to mental health scenarios. The selected mental health conditions covered a range of common and complex cases encountered in clinical settings. The LLMs were assessed for their prognosis of various mental health conditions, such as depression, suicidal thoughts, early schizophrenia, chronic schizophrenia, social phobia, and PTSD. Their evaluations were then compared to norms established by mental health professionals (general practitioners, psychiatrists, and psychologists), as reported by Morgan et al. (2013, 2014). Health professionals were selected based on their area of specialization to ensure a diverse representation of perspectives. The study evaluated four large language models (LLMs): Gemini, Claude, ChatGPT-3.5, and ChatGPT-4. Each model was tested 20 times across six clinical vignettes, including depression, suicidal ideation, early and chronic schizophrenia, social phobia, and PTSD, resulting in a total of 480 assessments. To explore gender-based variations, an additional set of 480 assessments was conducted (*n* = 480 male, *n* = 480 female), yielding a total of *N* = 960 evaluations.

### 2.2. Vignette Methodology

The study employed the text vignette methodology, utilizing six vignettes that represent different mental disorders (Reavley & Jorm, 2012). Each vignette was available in both male and female versions. Each vignette underwent ten separate assessments using various LLM tools. After the vignettes were input to LLMs, the models were asked a series of questions to evaluate identification, referral recommendations, and prognosis (Morgan et al., 2013).

All the vignettes depicted an individual with a mental disorder, with male participants referred to as ‘John’ and female participants referred to as ‘Mary’. The vignettes were designed to meet the diagnostic criteria outlined in the Diagnostic and Statistical Manual of Mental Disorders, 5th Edition (DSM-5) and the International Classification of Diseases, 11th Revision (ICD-11). The LLMs were asked to identify any issues with John/Mary that emerged from the vignettes and then to answer two questions assessing the perceived effectiveness of the various interventions. Question 1: ‘What is the most likely outcome for John/Mary given that he/she receives the type of professional help you deem most appropriate?’ (e.g., psychiatrist, psychologist, CBT, psychotherapy, antidepressants, antipsychotics). Question 2: ‘What is the most likely outcome for John/Mary if he/she does not receive any professional help?’ (with response options ranging from “Full recovery with no further problems” to “Condition will worsen”). Responses were assessed using a 6-point Likert scale, ranging from ‘full recovery with no further problems’ to ‘condition will worsen’ (Supplementary Materials).

The performance of all the LLMs was evaluated according to the criteria established by Morgan et al. (2013, 2014). Subsequently, we compared the LLMs’ performance to previously established norms derived from a sample of 1536 health professionals, including 518 general practitioners (GPs), 506 psychiatrists, 498 clinical psychologists (with 14 respondents not specifying their profession), and 6016 members of the Australian public.

### 2.3. Statistical Analyses

To determine the number of correct identifications for each LLM tool under each condition, string searches were applied to the LLMs’ open-ended answers to the question regarding the most appropriate diagnosis for the individual described in the vignette. Specifically, for the depression scenarios, any answer with the word ‘depress’ was counted. For depression with suicidal ideation, to be counted, an answer had to include both the string ‘depress’ and the string ‘suicid’ (to include responses containing words such as ‘suicidal’). For both early and chronic schizophrenia conditions, answers containing the string ‘schizophrenia’ or ‘psychosis’ were counted (after accounting for capital letters). Answers containing the string ‘social’ were counted for the social phobia condition, and answers that contained either the string ‘post’ or the string ‘trauma’ were counted for the PTSD condition. The obtained counts were calculated as percentages or counts for each statistical comparison and in accordance with the data provided in the articles (Morgan et al., 2013, 2014).

To provide a rigorous assessment of the effectiveness of various LLMs, the study utilized SPSS version 27 for statistical analysis. All the research hypotheses were examined using the χ2 test to identify differences. In instances where cell counts were too low, the Fisher’s exact test was used as an alternative. This methodological approach facilitated pattern quantification and underscored the potential of integrating LLMs into clinical decision-making. Chi-square tests were conducted, with significance level set at *p* < 0.01, reflecting a more conservative threshold. Additionally, Cramer’s V was used to measure effect sizes. Values ranged from 0 to 1, with 0.1, 0.3, and 0.5 representing small, medium, and large effect sizes, respectively. SPSS version 27 was used for chi-square tests and Fisher’s exact tests, while R software version 4.4.1 was utilized for generating advanced visualizations and calculating effect sizes (e.g., Cramer’s V). In addition to the automated analyses conducted using SPSS and R, manual validations were performed to cross-check the diagnostic outputs of the LLMs against predefined accuracy criteria. These validations ensured consistency and completeness in the responses analyzed.

## 3. Results

As noted, the detailed methodology examined four leading LLMs across a range of mental health conditions. This section describes the findings for diagnostic accuracy, treatment recommendations, and predicted outcomes. All analyses were conducted using R software version 4.4.1 (R Core Team, 2020) and RStudio version 2023.06.1 (R Core Team, 2023).

### 3.1. Comparison of Correct Diagnosis Rates for Different LLM Tools and Professionals

For both depression vignettes, the LLM tools (Gemini, ChatGPT-3.5, and ChatGPT-4) achieved a correct diagnosis rate of 100%, whereas the professionals exhibited a correct diagnosis rate of 95%. Fisher’s exact test yielded a *p*-value of 0.001 for both vignettes. For the vignette on depression and suicidal thoughts, ChatGPT-4 achieved a 100% correct diagnosis rate, outperforming the other models. Statistical analysis yielded a Cramer’s V of 0.81 with a *p*-value of <0.001, indicating significant differences. For the early schizophrenia vignette, ChatGPT-4 achieved a correct diagnosis rate of 55%, which was notably lower than the other entities, with a Cramer’s V of 0.55 and a *p*-value of <0.001. For the chronic schizophrenia vignette, professionals and ChatGPT-3.5 achieved similar correct diagnosis rates (95%), whereas ChatGPT-4 exhibited a lower rate (67%). Cramer’s V was 0.37 with a *p*-value of <0.001. For the social phobia and PTSD vignettes, all the LLM tools achieved a 100% correct diagnosis rate, outperforming professionals. Fisher’s exact test revealed significant differences, with *p*-values of less than 0.001 for both vignettes (Table 1).

### 3.2. Comparison of Treatments Across Different LLM Tools and Professionals

For each vignette, the treatment recommendations made by the LLMs were also compared to those of the various professionals. The therapies or activities chosen for the comparisons were based on the agreement of multiple practitioners (Haber et al., 2024; Bradford et al., 2024).

#### 3.2.1. Depression Vignette

Table 2 compares the treatment recommendations for depression made by GPs, psychiatrists, psychologists, and LLM tools (Claude, Gemini, ChatGPT-3.5, and ChatGPT-4). Notable disparities were observed between the groups. The LLM tools consistently recommended high rates of consulting a family doctor or GP (100%, except for ChatGPT-3.5 at 89.95%), surpassing GPs (95%) and psychiatrists (91%) as well as psychologists (76%). Recommendations to see a counselor were also higher among LLM tools (100%, except for Claude at 80%) than among GPs (86%), psychiatrists (49%), and psychologists (53%). Telephone counseling services were more frequently suggested by LLM tools (80–100%) than by professionals (57–79%). Recommendations to see a psychiatrist or psychologist were uniformly high, with LLM tools either matching or exceeding the recommendation rates of professionals. ChatGPT-4 exhibited the highest recommendation rate for antidepressants (100%), whereas the antidepressant recommendations of other LLM tools and professionals varied from 59% to 95.24%. LLM tools generally recommended activities and therapies at higher rates than did human professionals.

#### 3.2.2. Depression with Suicidal Thoughts Vignette

Table 3 compares treatment recommendations for depression with suicidal thoughts across various entities, including LLM tools (Claude, Gemini, ChatGPT-3.5, and ChatGPT-4) and mental health professionals. Significant differences were observed between the groups. Most LLM tools consistently recommended seeing a family doctor or GP (100%), with the exception of ChatGPT-3.5 (45%), while these recommendations among GPs, psychiatrists, and psychologists were 95%, 92%, and 83%, respectively. LLM tools also recommended seeing a counselor (100%), compared to GPs (86%), psychiatrists (47%), and psychologists (58%). Telephone counseling was universally recommended by LLM tools (100%) but less frequently by professionals (GPs—89%; psychiatrists—61%; and psychologists—83%). Psychiatrist consultations were recommended at high rates by all groups. LLM tools and professionals also varied in their recommendations for antidepressants and other interventions, with LLM tools generally advocating a broader range of activities and therapies.

#### 3.2.3. Early Schizophrenia Vignette

Recommendation rates for seeing a family doctor or GP were generally high: 100% for Gemini and 68% and 70%, respectively, for ChatGPT-3.5 and ChatGPT-4. Recommendations to see a family doctor were consistently high among GPs and psychiatrists (100% and 95%, respectively), whereas such recommendations among psychologists were somewhat lower (88%). Recommendations to see a social worker varied significantly, with LLM tools (Claude and ChatGPT-3.5) at 100%, compared to 65% for GPs, 32% for psychiatrists, and 37% for psychologists. All entities highly recommended seeing a psychiatrist (99% for psychologists and 100% for all other entities). The recommendation rate for seeing a psychologist was 100% for Gemini, with rates varying among the other LLM tools and professionals. Professionals and LLM tools also recommended antidepressants at rates ranging from 85% to 97%, except for Claude (30%) and Gemini (50%). Recommendations for cognitive behavior therapy and psychiatric admission exhibited substantial variability, with LLM tools generally advocating broader treatment options (Table 4).

#### 3.2.4. Chronic Schizophrenia Vignette

Significant differences were observed across LLM tools and professionals in their treatment recommendations for chronic schizophrenia. Most LLM tools and professionals highly recommended seeing a family doctor or GP, with Gemini at 94.44% and GPs and psychiatrists at 94% and 93%, respectively, whereas ChatGPT-3.5 and ChatGPT-4 exhibited lower rates (75% and 47.62%, respectively). Recommendations for consulting with social workers varied, with Gemini at 100% and others exhibiting lower rates: 74% (GPs), 58% (psychiatrists), and 72% (psychologists). All entities consistently recommended seeing a psychiatrist (95.24–100%). Recommendations to see a psychologist were highest for Gemini (100%) and lower for others. Antidepressants were strongly recommended by professionals (94–99%), while LLM tools showed more variability. Recommendations for cognitive behavior therapy and psychiatric ward admission also varied, with ChatGPT-4 exhibiting higher rates. Recommendations for physical activity, alcohol consumption, and information from health educators varied more across entities (Table 5).

#### 3.2.5. Social Phobia Vignette

In the social phobia condition, significant differences were observed across all entities. The LLM tools recommended seeing a family doctor or GP at a rate of 100%, except for ChatGPT-4 (76.19%). In contrast, 91% of GPs, 71% of psychiatrists, and 64% of psychologists recommended seeing a family doctor in the case of social phobia. The LLM tools also consistently recommended seeing a counselor (100%), whereas these recommendations were lower among professionals: 87% (GPs), 57% (psychiatrists), and 49% (psychologists). Telephone counseling services were highly recommended by LLM tools (85.71–100%), with lower recommendation rates among professionals (35–76%). Seeing a psychiatrist was universally recommended by LLM tools, whereas the recommendations of professionals varied. Antidepressants were recommended at varying rates, with ChatGPT-4 at 95.24% and lower rates for other entities. LLM tools generally recommended activities such as physical exercise and social engagement at higher rates than professionals. Cognitive behavior therapy and psychotherapy were also recommended more frequently by the LLM tools (Table 6).

#### 3.2.6. Post-Traumatic Stress Disorder Vignette

In the PTSD condition, significant differences were observed among all entities. Most LLM tools highly recommended seeing a family doctor or GP (90–100%), whereas the recommendations of the professionals were lower: 93% (GPs), 78% (psychiatrists), and 69% (psychologists). The LLM tools also consistently recommended seeing a counselor for PTSD (100%), whereas the professional recommendations were lower: 80% (GPs), 38% (psychiatrists), and 49% (psychologists). Telephone counseling was also highly recommended by the LLM tools (90–100%) and less recommended by the professionals: 69% (GPs), 47% (psychiatrists), and 62% (psychologists). Seeing a psychiatrist was universally recommended by the LLM tools, with the professionals also exhibiting high rates. Recommendations for seeing a psychologist for PTS varied, with the LLM tools ranging from 60% to 100% and the professionals showing slightly higher consistency. Antidepressant recommendations were higher among LLM tools such as ChatGPT-3.5 and ChatGPT-4 (90%), whereas the professionals’ rates of antidepressant recommendation were lower. Activities and therapies, including physical activity and relaxation techniques, were highly recommended by the LLM tools, often at higher rates than the professionals (Table 7).

**Figure 1:**
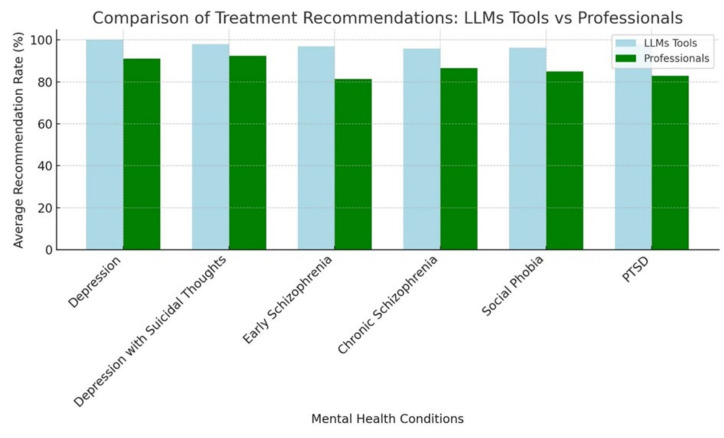
Comparison of average treatment recommendations by LLM tools and mental health professionals across mental health conditions.

Overall, this study revealed key differences in treatment recommendations between LLM tools and mental health professionals. The LLM tools consistently recommended family doctors, counselors, and telephone counseling more frequently across conditions such as depression and PTSD. The tools also suggested a broader range of treatments, including antidepressants and therapeutic activities, especially in cases of social phobia and schizophrenia. Professionals showed more consistency in recommending psychiatric consultations and specific medications, whereas LLM tools emphasized a wider array of treatments. Overall, LLM tools were more proactive in recommending diverse options than human professionals (Figure 1).

### 3.3. Comparison Between LLM Tools and Mental Health Professionals Regarding Predicted Outcomes for Those Who Receive Help and Those Who Do Not

The study also compared outcome predictions across various mental health conditions as described in the vignettes, both for those who received professional assistance and for those who did not.

For individuals who received help (Table 8), no significant differences in outcome predictions were found between professionals and LLM tools. Nevertheless, the professionals consistently demonstrated a more optimistic outlook regarding full recovery rates. For instance, in cases of depression with suicidal thoughts, professionals predicted a full recovery rate of 94.4%, whereas the predictions of the LLM tools varied widely, with Claude at 55% and ChatGPT-4 at 13.64%. Partial recovery predictions also differed, with professionals estimating 5.2% and LLM tools such as Gemini and ChatGPT-4 predicting 66.67% and 86.36%, respectively.

For early schizophrenia, professionals predicted a 60.4% full recovery rate compared with a 5% recovery rate predicted by Claude. For chronic schizophrenia, professionals predicted a 20.1% full recovery rate, whereas ChatGPT-4 predicted a 0% recovery rate. Social phobia and PTSD also exhibited significant differences, with LLM tools generally predicting lower full recovery rates and higher partial recovery rates than professionals. These discrepancies highlight the variability in LLM predictions and the generally optimistic outlook of human professionals.

For those who did not receive help (Table 9), the outlook for recovery was significantly worse across all conditions and entities. Professionals predicted a 7.7% full recovery rate for depression, with LLM tools predicting a 0% recovery rate. For early schizophrenia, no entities predicted full recovery, and all exhibited very low partial recovery rates. For chronic schizophrenia and social phobia, no full recovery was predicted, and partial recovery rates were low across the board. PTSD predictions followed similar trends, with professionals predicting some recovery but LLM tools often predicting none. These findings underscore the generally more negative outlook of LLM tools compared with human professionals, especially when no professional help is provided.

**Figure 2:**
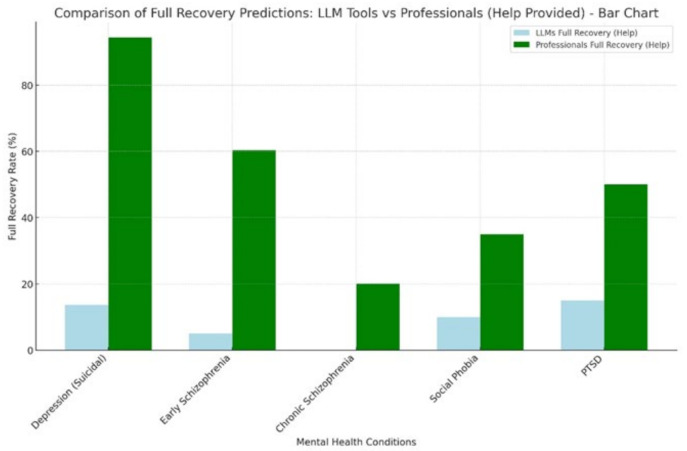
Comparison of full recovery predictions: LLM tools vs. professionals (help provided).

For those who received help, professionals consistently predicted higher full recovery rates than did LLM tools, particularly in cases of depression with suicidal thoughts (94.4% vs. lower rates among LLMs). For conditions such as early and chronic schizophrenia, professionals also showed a more optimistic outlook than LLM tools, which predicted minimal rates of recovery (Figure 2). For those who did not receive help, both LLM tools and professionals predicted worse outcomes, with professionals maintaining a slightly more optimistic view. Overall, LLM tools were more cautious in their predictions, especially when no professional assistance was involved.

## 4. Discussion

This study sought to compare various LLM tools and mental health professionals with respect to diagnosis accuracy, recommended treatments, and predicted outcomes in the case of different mental health conditions. Individuals who received help were compared with those who did not.

In the present study, all LLM tools achieved a 100% correct diagnosis rate for the depression vignettes, while professionals recorded a slightly lower accuracy rate of 95%. ChatGPT-4 demonstrated superior performance for the depression with suicidal thoughts vignette, achieving a 100% correct diagnosis rate and significantly outperforming the other entities. Yet ChatGPT-4 exhibited a notably lower correct diagnosis rate of 55% for early schizophrenia, compared to the 95–100% rates achieved by the other entities. Similarly, ChatGPT-4 underperformed in the chronic schizophrenia vignette, with a 67% correct diagnosis rate compared to the 95% rate achieved by professionals. Despite these shortcomings, all the LLM tools consistently achieved a 100% correct diagnosis rate for the social phobia and PTSD vignettes, surpassing the performance of professionals.

The observed underperformance of ChatGPT-4 in diagnosing early schizophrenia and chronic schizophrenia highlights a potential limitation in the ability of current LLMs to generalize across diverse mental health conditions (Guo et al., 2024). This variability suggests that while LLMs are effective in diagnosing common conditions such as depression (Elyoseph & Levkovich, 2023; Weisenburger et al., 2024), their reliability may diminish when faced with more complex or less common disorders (Levkovich & Omar, 2024). A study analyzing approximately 3000 posts from clinicians regarding the ethical concerns of using LLMs in healthcare raised a number of key issues, including the fairness and reliability of these systems, as well as concerns over data accuracy (Mirzaei et al., 2024). These findings underscore the importance of continuous model improvement and the need for caution when relying solely on LLMs to diagnose complex cases (Lawrence et al., 2024). The mixed performance of ChatGPT-4 across different conditions further emphasizes the necessity for additional training and evaluation of LLMs (Stade et al., 2024). Ensuring that these tools are trained on diverse and representative datasets is crucial for enhancing their generalizability and reliability across a broad spectrum of mental health conditions (Cabrera et al., 2023). Moreover, ongoing evaluation in real-world clinical settings is essential, both to ensure that these tools consistently perform at a high level and to provide accurate diagnostic support (Ohse et al., 2024).

In the current study, significant disparities in treatment recommendations were observed between LLM tools and professionals across all vignettes. For the depression vignette, LLM tools consistently recommended seeing a GP (100% rate), exceeding the recommendations made by professionals. Similar trends were noted for counselor recommendations, with LLM tools showing greater consistency. In contrast, professionals demonstrated more variability in recommending antidepressants, whereas ChatGPT-4 consistently recommended them (100% rate). In the depression with suicidal thoughts vignette, LLM tools frequently recommended a broader range of treatment options, including high recommendation rates for physical activities and psychotherapy. Across all vignettes, LLM tools generally and more frequently advocate for a wider range of treatments, including physical exercise and cognitive behavioral therapy, than professionals did.

The consistent recommendation patterns observed in LLM tools, such as the nearly universal suggestion to see a GP (100% across various vignettes), indicate that these tools may be programmed to prioritize certain baseline interventions (Golden et al., 2024). In a study involving three hypothetical patient scenarios with significant complaints, ChatGPT was used as a virtual assistant to a psychiatrist (Dergaa et al., 2024). While ChatGPT’s initial recommendations were appropriate, as the complexity of the clinical cases increased, the recommendations became inappropriate and potentially dangerous in some instances (Dergaa et al., 2024). The lack of variability in these recommendations may also indicate a limitation in the ability of these tools to tailor advice to specific patient needs, potentially leading to overgeneralization (Higgins et al., 2023). This limitation highlights a deficiency in the models’ capacity for clinical judgment and nuanced decision-making (Dergaa et al., 2024). To maintain the quality of patient care, it is crucial to use LLMs as complementary tools rather than as replacements for professional expertise (Prabhod, 2023; Xie et al., 2024).

In the present study, LLM tools consistently predicted less optimistic mental health outcomes for individuals receiving help than did professionals. For depression with suicidal thoughts, Claude predicted a 55% full recovery rate and ChatGPT-4 only a 13.64% full recovery rate, whereas professionals estimated the full recovery rate at 94.4%. For early schizophrenia, Claude predicted a 5% full recovery, in contrast to professionals’ prediction of 60.4%. LLMs often predicted no recovery for untreated individuals across various conditions, whereas professionals generally expected some level of improvement. Additionally, LLMs tend to predict lower rates of full recovery and higher rates of partial recovery than professional assessments. The complexity of prognosis prediction is also evident in studies incorporating extensive personal and medical information. For example, a review of 30 studies analyzed the use of AI methods to predict clinical outcomes in patients with psychotic disorders, where detailed patient histories, including medical records and personal factors, were given. The results revealed predicted accuracy ranging from 48% to 89% for the AI methods (Tay et al., 2024).

The consistent optimism of human professionals regarding full recovery rates highlights the importance of clinical experience and judgment in mental health treatment (Leamy et al., 2023). Professionals’ positive outlooks may reflect a broader understanding of patient resilience, therapeutic potential, and the nuances of mental health conditions, which LLM tools currently lack (Alhuwaydi, 2024). This suggests that while LLMs can be valuable in supporting diagnosis and treatment planning, they should not replace the nuanced judgment of experienced clinicians (Alowais et al., 2023).

The variability and conservative predictions of LLM tools, especially in cases in which professional intervention is lacking, raise concerns about their reliability and ethical use in mental health (Hadar-Shoval et al., 2024; Mirzaei et al., 2024). These tools often display a pessimistic bias, which may underestimate recovery potential and negatively affect treatment planning if used without professional oversight (Weisenburger et al., 2024). This finding underscores the importance of carefully integrating LLMs into clinical practice to support, rather than hinder, patient outcomes (Elyoseph & Levkovich, 2024; Golden et al., 2024). Continued refinement of LLMs that incorporate diverse real-world clinical data is necessary to improve their accuracy (Aich et al., 2024). Furthermore, combining LLM tools with ongoing professional input can address the current limitations, thus reinforcing the irreplaceable role of human clinical expertise in mental healthcare (Lawrence et al., 2024).

### Limitations

This study utilized valid vignettes employed in previous research across a range of professionals and mental disorders. Nevertheless, these vignettes are not real cases, and the use of text-based scenarios may not fully capture the complexities of real-life patient interactions or the broader spectrum of mental health conditions, thereby limiting the generalizability of the findings. Additionally, the study acknowledges the inherent biases within LLMs, which are trained on extensive datasets that may contain biases influencing their diagnostic and treatment recommendations. The ‘black box’ nature of LLMs further complicates this issue, making it challenging to discern the rationale behind specific recommendations or diagnoses—a crucial factor in clinical contexts for establishing trust and ethical practice. The findings of this study are based on comparisons with health professionals, yet they lack direct clinical validation in actual patient care, underscoring the need for future research to focus on clinical trials and real-world applications to assess the efficacy and safety of LLMs in mental health diagnostics and treatment planning. Moreover, given the rapid development of AI technologies, the capabilities of LLMs are continuously evolving, such that newer versions of the models assessed in this study may perform differently, highlighting the necessity for ongoing evaluation. Addressing these limitations provides a more comprehensive understanding of the challenges and considerations in applying LLM technology to mental health, paving the way for more informed and ethical research and its implementation in the future.

## 5. Conclusions

This study compared the diagnostic accuracy and treatment recommendations of Gemini, Claude, ChatGPT-3.5, and ChatGPT-4 with those of mental health professionals for various mental health conditions. Text vignettes were used to evaluate the performance of LLMs and compare it to norms established by a sample of health professionals. The LLMs demonstrated high diagnostic accuracy, with 100% correct diagnosis rates for depression, social phobia, and PTSD, often surpassing professionals. However, ChatGPT-4 was less accurate in the case of early and chronic schizophrenia than other entities. The LLMs consistently recommended consulting healthcare professionals at higher rates than the professionals themselves.

The LLMs exhibited more conservative estimates, generally predicting lower and higher rates of full and partial recovery, respectively. Conversely, human experts consistently demonstrated a more optimistic outlook regarding full recovery across various conditions, including depression, suicidal ideation, schizophrenia, and PTSD. While both groups forecasted poorer outcomes in the absence of intervention, the LLMs displayed a notably more pessimistic perspective. These findings underscore the contrast between the generally hopeful prognoses of human professionals and the more cautious predictions of LLMs in the context of mental health recovery. The results highlight the potential for integrating LLMs into clinical decision-making processes; however, further research is necessary to validate these findings and overcome the study’s limitations.

## Figures and Tables

**Table 1:** Comparison of correct diagnosis rates across different LLM tools and professionals.

Vignette	Professionals	Claude	Gemini	ChatGPT-3.5	ChatGPT-4	Cramer’s V	*p*
Depression	95%	95%	100%	100%	100%		0.001
Depression and suicidal thoughts	12%	10%	11%	5%	100%	0.81	<0.001
Early schizophrenia	95%	95%	100%	100%	55%	0.55	<0.001
Chronic schizophrenia	95%	85%	100%	95%	67%	0.37	<0.001
Social phobia	86%	100%	100%	100%	100%		<0.001
PTSD	90%	95%	100%	100%	100%		<0.001

**Table 2:** Comparison of treatments for depression across different entities.

Depression	GP	Psychiatrists	Psychologists	Claude	Gemini	GPT 3.5	GPT 4	Cramer’s V	*p*
People									
A typical family GP or doctor	95	91	76	100	100	80.95	100	0.33	<0.001
A counsellor	86	49	53	80	100	100	100	0.52	<0.001
Telephone counselling service (e.g., Lifeline)	79	57	63	80	100	100	85.71	0.39	<0.001
A psychiatrist	92	92	75	100	100	100	100	0.36	<0.001
A psychologist	93	86	93	75	100	95.24	100	0.3	<0.001
Medications									
Antidepressants	80	80	59	60	68.42	95.24	100	0.36	<0.001
Activities/therapies									
Becoming more physically active	90	95	93	50	100	95.24	100	0.52	<0.001
Reading about people with similar problems and how they have dealt with them	83	82	66	60	100	95.24	95.24	0.38	<0.001
Getting out and about more	83	70	80	85	100	95.24	100	0.32	<0.001
Courses on relaxation, stress management, meditation or yoga	79	69	72	80	100	95.24	100	0.34	<0.001
Cutting out alcohol altogether	66	82	62	90	100	95.24	100	0.41	<0.001
Psychotherapy	82	73	73	75	100	95.24	100	0.33	<0.001
Cognitive behaviour therapy	89	82	90	60	63.16	90.48	90.48	0.31	<0.001
Consulting a website that gives information about his/her problem	74	64	60	50	94.74	80.95	80.95	0.31	<0.001
Consulting an expert using email or the web about his/her problem	63	60	64	90	100	80.95	85.71	0.35	<0.001
Consulting a book that gives information about his/her problem	76	66	67	75	94.74	61.9	85.71	0.25	<0.001
Receiving information about the problem from a health educator	88	69	74	75	78.95	66.67	90.48	0.2	<0.001

Note: data given in %, GPs—general practitioners.

**Table 3:** Comparison of treatments for depression with suicidal thoughts across different entities.

Depression with Suicidal Thoughts	GP	Psychiatrists	Psychologists	Claude	Gemini	GPT 3.5	GPT 4	Cramer’s V	*p*
People									
A typical family GP or doctor	95	92	83	100	100	45	100	0.56	<0.001
A counsellor	86	47	58	100	94.44	100	100	0.55	<0.001
Telephone counselling service (e.g., Lifeline)	89	61	83	100	100	100	100	0.46	<0.001
A psychiatrist	92	95	83	100	100	100	100	-	<0.001
A psychologist	93	79	98	100	94.44	95	100	0.29	<0.001
Medications									
Antidepressants	95	90	72	95	100	95	95.45	0.31	<0.001
Activities/therapies									
Becoming more physically active	92	83	96	60	100	100	95.45	0.43	<0.001
Reading about people with similar problems and how they have dealt with them	80	81	75	100	94.44	95	72.73	0.28	<0.001
Getting out and about more	77	64	87	100	100	95	95.45	0.39	<0.001
Courses on relaxation, stress management, meditation or yoga	85	61	87	100	94.44	95	100	0.4	<0.001
Cutting out alcohol altogether	85	74	71	100	88.89	85	100	0.31	<0.001
Psychotherapy	83	81	82	100	94.44	95	100	0.27	<0.001
Cognitive behaviour therapy	91	79	89	90	94.44	95	100	0.21	<0.001
Consulting a website that gives information about his/her problem	66	61	66	100	94.44	100	72.73	0.4	<0.001
Consulting an expert using email or the web about his/her problem	51	55	72	100	100	95	72.73	0.46	<0.001
Consulting a book that gives information about his/her problem	63	55	71	90	94.44	75	77.27	0.3	<0.001
Receiving information about the problem from a health educator	86	64	76	60	72.22	80	77.27	0.19	<0.001

Note: data given in %, GPs—general practitioners.

**Table 4:** Comparison of treatments for early schizophrenia across different entities.

Early Schizophrenia	GP	Psychiatrists	Psychologists	Claude	Gemini	GPT 3.5	GPT 4	Cramer’s V	*p*
People									
A typical family GP or doctor	100	95	88	80	100	68	70	0.36	<0.001
A social worker	65	32	37	100	94.44	100	70	0.59	<0.001
A psychiatrist	100	100	99	100	100	100	100	-	1
A psychologist	86	58	92	65	100	75	80	0.34	<0.001
Medications									
Antidepressants	91	97	88	30	50	85	85	0.54	<0.001
Activities/therapies									
Becoming more physically active	65	52	67	80	94.44	100	80	0.37	<0.001
Cutting out alcohol altogether	84	71	76	80	11.11	50	65	0.48	<0.001
Cognitive behaviour therapy	45	32	80	55	94.44	55	100	0.5	<0.001
Admission to a psychiatric ward of a hospital	76	78	59	5	16.67	30	95	0.64	<0.001
Receiving information about the problem from a health educator	64	58	75	80	72.22	55	55	0.2	<0.001

Note: data given in %, GPs—general practitioners.

**Table 5:** Comparison of treatments for chronic schizophrenia across different entities.

Early Schizophrenia	GP	Psychiatrists	Psychologists	Claude	Gemini	GPT 3.5	GPT 4	Cramer’s V	*p*
People									
A typical family GP or doctor	94	93	83	70	94.44	75	47.62	0.39	<0.001
A social worker	74	58	72	35	100	90	52.38	0.45	<0.001
A psychiatrist	98	98	96	100	100	90	95.24	-	<0.001
A psychologist	78	57	86	55	100	55	71.43	0.36	<0.001
Medications									
Antidepressants	96	99	94	90	22.22	45	57.14	0.63	<0.001
Activities/therapies									
Becoming more physically active	53	70	66	90	61.11	75	38.1	0.32	<0.001
Cutting out alcohol altogether	49	52	38	85	44.44	60	38.1	0.3	<0.001
Cognitive behaviour therapy	35	41	71	50	83.33	55	90.48	0.4	<0.001
Admission to a psychiatric ward of a hospital	75	85	62	65	5.56	25	95.24	0.61	<0.001
Receiving information about the problem from a health educator	59	63	65	80	50	20	28.57	0.39	<0.001

Note: data given in %, GPs—general practitioners.

**Table 6:** Comparison of treatments for chronic social phobia across different entities.

Social Phobia	GP	Psychiatrists	Psychologists	Claude	Gemini	GPT 3.5	GPT 4	Cramer’s V	*p*
People									
A typical family GP or doctor	91	71	64	100	100	85	76.19	0.36	<0.001
A counsellor	87	57	49	100	83.33	100	100	0.52	<0.001
Telephone counselling service (e.g., Lifeline)	76	44	35	100	94.44	90	85.71	0.54	<0.001
A psychiatrist	85	96	70	100	100	90	100	0.37	<0.001
A psychologist	94	90	96	100	77.78	60	95.24	0.39	<0.001
Medications									
Antidepressants	57	72	41	90	72.22	65	95.24	0.38	<0.001
Activities/therapies									
Becoming more physically active	84	79	83	95	100	95	95.24	0.25	<0.001
Reading about people with similar problems and how they have dealt with them	83	85	88	100	100	100	100	0.3	<0.001
Getting out and about more	63	72	79	100	100	95	100	0.43	<0.001
Courses on relaxation, stress management, meditation or yoga	89	90	90	100	100	80	100	0.27	<0.001
Cutting out alcohol altogether	50	44	42	100	38.89	60	95.24	0.49	<0.001
Psychotherapy	86	77	74	90	100	65	95.24	0.31	<0.001
Cognitive behaviour therapy	93	96	98	50	88.89	65	100	0.49	<0.001
Consulting a website that gives information about his/her problem	64	75	78	100	100	45	100	0.49	<0.001
Consulting an expert using email or the web about his/her problem	60	71	74	100	100	70	100	0.42	<0.001
Consulting a book that gives information about his/her problem	66	74	78	100	100	55	100	0.44	<0.001
Receiving information about the problem from a health educator	85	79	80	100	94.44	30	100	0.57	<0.001

Note: data given in %, GPs—general practitioners.

**Table 7:** Comparison of treatments for post-traumatic stress disorder across different entities.

Post-Traumatic Stress Disorder (PTSD)	GP	Psychiatrists	Psychologists	Claude	Gemini	GPT 3.5	GPT 4	Cramer’s V	*p*
People									
A typical family GP or doctor	93	78	69	90	100	90	95	0.3	<0.001
A counsellor	80	38	49	100	88.89	100	100	0.59	<0.001
Telephone counselling service (e.g., Lifeline)	69	47	62	100	100	95	90	0.49	<0.001
A psychiatrist	92	96	74	100	100	100	100	0.39	<0.001
A psychologist	96	81	99	60	72.22	65	100	0.4	<0.001
Medications									
Antidepressants	67	81	38	50	50	90	90	0.41	<0.001
Activities/therapies									
Becoming more physically active	87	82	79	50	100	100	95	0.45	<0.001
Reading about people with similar problems and how they have dealt with them	91	73	81	90	100	100	95	0.31	<0.001
Getting out and about more	67	58	65	100	100	85	100	0.45	<0.001
Courses on relaxation, stress management, meditation or yoga	82	70	81	100	94.44	75	100	0.32	<0.001
Cutting out alcohol altogether	63	66	55	95	61.11	60	100	0.37	<0.001
Psychotherapy	86	76	76	70	100	60	100	0.36	<0.001
Cognitive behaviour therapy	97	81	91	95	94.44	80	95	0.22	<0.001
Consulting a website that gives information about his/her problem	55	60	79	75	94.44	65	95	0.34	<0.001
Consulting an expert using email or the web about his/her problem	55	51	72	75	100	70	95	0.39	<0.001
Consulting a book that gives information about his/her problem	57	63	75	75	100	50	95	0.39	<0.001
Receiving information about the problem from a health educator	79	68	78	70	77.78	65	95	0.22	<0.001

Note: data given in %, GPs—general practitioners.

**Table 8:** Predicted outcome for those who receive help.

	Vignette	Professionals	Claude	Gemini	GPT 3.5	GPT 4	*p*
Full recovery	Depression	97	25	84.21	0	0	1
Partial recovery	Depression	3	75	15.79	100	100	
No improvement	Depression	0	0	0	0	0	
Get worse	Depression	0	0	0	0	0	
Full recovery	Depression with suicidal thoughts	94.4	55	33.33	0	13.64	0.14
Partial recovery	Depression with suicidal thoughts	5.2	45	66.67	100	86.36	
No improvement	Depression with suicidal thoughts	0.4	0	0	0	0	
Get worse	Depression with suicidal thoughts	0	0	0	0	0	
Full recovery	Early schizophrenia	60.4	5	0	0	0	0.14
Partial recovery	Early schizophrenia	38.7	95	94.44	100	100	
No improvement	Early schizophrenia	0.9	0	0	0	0	
Get worse	Early schizophrenia	0	0	5.56	0	0	
Full recovery	Chronic schizophrenia	20.1	20	5.56	0	0	1
Partial recovery	Chronic schizophrenia	78	80	44.44	95	100	
No improvement	Chronic schizophrenia	2	0	38.89	5	0	
Get worse	Chronic schizophrenia	0	0	11.11	0	0	
Full recovery	Social phobia	65	20	11.11	0	0	0.14
Partial recovery	Social phobia	34.6	80	88.89	100	100	
No improvement	Social phobia	0.4	0	0	0	0	
Get worse	Social phobia	0	0	0	0	0	
Full recovery	PTSD	89.6	35	22.22	0	15	1
Partial recovery	PTSD	10	65	77.78	100	85	
No improvement	PTSD	0.4	0	0	0	0	
Get worse	PTSD	0.2	0	0	0	0	

Note: data given in %, GPs—general practitioners.

**Table 9:** Predicted outcome for those who don’t receive help.

	Vignette	Professionals	Claude	Gemini	GPT 3.5	GPT 4	*p*
Full recovery	Depression	7.7	0	0	0	0	1
Partial recovery	Depression	35.8	25	31.58	33.33	4.76	
No improvement	Depression	9.8	55	10.53	57.14	38.1	
Get worse	Depression	46.7	20	57.89	9.52	57.14	
Full recovery	Depression with suicidal thoughts	4.7	0	0	0	0	1
Partial recovery	Depression with suicidal thoughts	20	10	0	0	0	
No improvement	Depression with suicidal thoughts	10.6	35	0	75	0	
Get worse	Depression with suicidal thoughts	64.7	55	100	25	100	
Full recovery	Early schizophrenia	0	0	0	0	0	1
Partial recovery	Early schizophrenia	4.3	0	0	15	0	
No improvement	Early schizophrenia	6.8	45	0	55	0	
Get worse	Early schizophrenia	88.9	55	100	30	100	
Full recovery	Chronic schizophrenia	0	0	0	0	0	1
Partial recovery	Chronic schizophrenia	2	0	0	0	9.52	
No improvement	Chronic schizophrenia	29	60	0	45	4.76	
Get worse	Chronic schizophrenia	69	40	100	55	85.71	
Full recovery	Social phobia	0	0	0	0	0	1
Partial recovery	Social phobia	10.9	40	11.11	5	4.76	
No improvement	Social phobia	32.2	55	33.33	95	42.86	
Get worse	Social phobia	57	5	55.56	0	52.38	
Full recovery	PTSD	6.9	0	0	0	0	1
Partial recovery	PTSD	44.3	35	16.67	20	10	
No improvement	PTSD	13.4	45	5.56	65	15	
Get worse	PTSD	35.4	20	77.78	15	75	

Note: data given in %, GPs—general practitioners.

## Data Availability

The data that support the findings of this study are available from the corresponding author, I.L., upon request.

## Supplementary Materials

The following supporting information can be downloaded at https://www.mdpi.com/article/10.3390/ejihpe15010009/s1.
