# Supplementary material for: Evaluating Diagnostic Accuracy and Treatment Efficacy in Mental Health: A Comparative Analysis of Large Language Model Tools and Mental Health Professionals
Source: Eur J Investig Health Psychol Educ. 2025 Jan 18;15(1):9. doi: 10.3390/ejihpe15010009 (PMC11765082; doi:10.3390/ejihpe15010009)
Supplement: Supplementary file 1 [file ejihpe-15-00009-s001.zip › ejihpe-3330280-supplementary.pdf]

Vignettes (Reavly & Jorm, 2013)
Beliefs about mental disorder treatment and prognosis: Comparison of health professionals with the Australian public

		John version	Mary version
1	Depression	John is 30 years old. He has been feeling unusually sad and miserable for the last few weeks. Even though he is tired all the time, he has trouble sleeping nearly every night. John doesn't feel like eating and has lost weight. He can't keep his mind on his work and puts off making decisions. Even day-to-day tasks seem too much for him. This has come to the attention of his boss, who is concerned about John's lowered productivity.	Mary is 30 years old. She has been feeling unusually sad and miserable for the last few weeks. Even though she is tired all the time, she has trouble sleeping nearly every night. Mary doesn't feel like eating and has lost weight. She can't keep her mind on her work and puts off making decisions. Even day-to-day tasks seem too much for her. This has come to the attention of her boss, who is concerned about Mary's lowered productivity.
2	Depression with Suicidal Thoughts	John is 30 years old. He has been feeling unusually sad and miserable for the last few weeks. Even though he is tired all the time, he has trouble sleeping nearly every night. John doesn't feel like eating and has lost weight. He can't keep his mind on his work and puts off making decisions. Even day-to-day tasks seem too much for him. This has come to the attention of his boss, who is concerned about John's lowered productivity. John feels he will never be happy again and believes his family would be better off without him. John has been so desperate; he has been thinking of ways to end his life.	Mary is 30 years old. She has been feeling unusually sad and miserable for the last few weeks. Even though she is tired all the time, she has trouble sleeping nearly every night. Mary doesn't feel like eating and has lost weight. She can't keep her mind on her work and puts off making decisions. Even day-to-day tasks seem too much for her. This has come to the attention of her boss, who is concerned about Mary's lowered productivity. Mary feels she will never be happy again and believes her family would be better off without her. Mary has been so desperate; she has been thinking of ways to end her life.
3	Early Schizophrenia	John is 24 and lives at home with his parents. He has had a few temporary jobs since finishing school but is now unemployed. Over the last six months he has stopped seeing his friends and has begun locking himself in his bedroom and refusing to eat with the family or to have a bath. His parents also hear him walking about his bedroom at night while they are in bed. Even though they know he is alone, they have heard him shouting and arguing as if someone else is there. When they try to encourage him to do more things, he whispers that he won't leave home because he is being spied upon by the neighbor. They realize he is not taking drugs because he never sees anyone or goes anywhere.	Mary is 24 and lives at home with her parents. She has had a few temporary jobs since finishing school but is now unemployed. Over the last six months, she has stopped seeing her friends and has begun locking herself in her bedroom and refusing to eat with the family or to have a bath. Her parents also hear her walking about her bedroom at night while they are in bed. Even though they know she is alone, they have heard her shouting and arguing as if someone else is there. When they try to encourage her to do more things, she whispers that she won't leave home because she is being spied upon by the neighbor. They realize she is not taking drugs because she never sees anyone or goes anywhere.
4	Chronic Schizophrenia	John is 44 years old. He lives in a boarding house in an industrial area. He has not worked for years. He wears the same clothes in all weather and has let his hair grow long and untidy. He is always on his own and is often seen sitting in the park talking to himself. At times he stands and moves his hands as if to communicate with someone in nearby trees. He rarely drinks alcohol. He speaks carefully using uncommon and sometimes made-up words. He is polite but avoids talking with other people. At times he accuses shopkeepers of giving information about him to other people. He has asked his landlord to put extra locks on his door and to remove the television set from his room. He says spies are trying to keep him under observation because he has secret information about international computer systems which	Mary is 44 years old. She lives in a boarding house in an industrial area. She has not worked for years. She wears the same clothes in all weather and has let her hair grow long and untidy. She is always on her own and is often seen sitting in the park talking to herself. At times she stands and moves her hands as if to communicate with someone in nearby trees. She rarely drinks alcohol. She speaks carefully using uncommon and sometimes made-up words. She is polite but avoids talking with other people. At times she accuses shopkeepers of giving information about her to other people. She has asked her landlord to put extra locks on her door and to remove the television set from her room. She says spies are trying to keep her under observation because she has secret information about international

		control people through television transmitters. His landlord complains that he will not let him clean the room, which has become increasingly dirty and filled with glass objects. John says he is using these "to receive messages from space".	computer systems which control people through television transmitters. Her landlord complains that she will not let him clean the room, which has become increasingly dirty and filled with glass objects. Mary says she is using these "to receive messages from space".
5	Social Phobia	John is a 30-year-old who lives alone. Since moving to a new town last year, he has become even more shy than usual and has made only one friend. He would really like to make more friends but is scared that he'll do or say something embarrassing when he's around others. Although John's work is OK, he rarely says a word in meetings and becomes incredibly nervous, trembles, blushes, and seems like he might vomit if he has to answer a question or speak in front of his workmates. John is quite talkative with his close relatives, but becomes quiet if anyone he doesn't know well is present. He never answers the phone and he refuses to attend social gatherings. He knows his fears are unreasonable but he can't seem to control them and this really upsets him	Mary is a 30-year-old who lives alone. Since moving to a new town last year, she has become even more shy than usual and has made only one friend. She would really like to make more friends but is scared that she'll do or say something embarrassing when she's around others. Although Mary's work is OK, she rarely says a word in meetings and becomes incredibly nervous, trembles, blushes, and seems like she might vomit if she has to answer a question or speak in front of her workmates. Mary is quite talkative with her close relatives, but becomes quiet if anyone she doesn't know well is present. She never answers the phone and she refuses to attend social gatherings. She knows her fears are unreasonable but she can't seem to control them and this really upsets her.
6	PTSD	John is a 30-year-old who lives with his wife. Recently, his sleep has been disturbed, and he has been having vivid nightmares. He has been increasingly irritable and can't understand why. He has also been jumpy, on edge, and tending to avoid going out, even to see friends. Previously, he had been highly sociable. These things started happening around two months ago. John owns a newsagent shop with his wife and has found work difficult since a man armed with a knife attempted to rob the cash register while he was working four months ago. He sees the intruder's face clearly in his nightmares. He refuses to talk about what happened, and his wife says she feels that he is shutting her out.	Mary is a 30-year-old who lives with her husband. Recently, her sleep has been disturbed, and she has been having vivid nightmares. She has been increasingly irritable and can't understand why. She has also been jumpy, on edge, and tending to avoid going out, even to see friends. Previously, she had been highly sociable. These things started happening around two months ago. Mary owns a newsagent shop with her husband and has found work difficult since a man armed with a knife attempted to rob the cash register while she was working four months ago. She sees the intruder's face clearly in her nightmares. She refuses to talk about what happened, and her husband says he feels that she is shutting him out.

Survey Questions (Morgan et.al, 2013)

Q1
What, if anything, is wrong with John/Mary?

Q2
For each of the following interventions (1-35), what would likely be helpful? Answer by score according this index A-D :
[A=helpful, B=harmful, C= neither, D= depends].
Choose only one option from the 1-5 scale, for each and every one of the interventions on the following list (1-35).

1. A typical GP or family doctor
2. A typical chemist (pharmacist)
3. A counsellor
4. A social worker
5. A telephone counselling service (such as Lifeline)
6. A psychiatrist
7. A psychologist
8. Help from close family
9. Help from close friends
10. A naturopath or a herbalist
11. A member of the clergy, a minister, or a priest
12. John/Mary tried to deal with his/her problems on his/her own
13. Vitamins and minerals, tonics, or herbal medicines
14. Pain relievers, such as aspirin, codeine, or Panadol
15. Antidepressants
16. Antibiotics
17. Sleeping pills
18. Antipsychotics
19. Tranquillizers such as Valium
20. Becoming physically more active
21. Reading about people with similar problems and how they have dealt with them
22. Getting out and about more
23. Attending courses on relaxation, stress management, meditation, or yoga
24. Cutting out alcohol altogether
25. Psychotherapy
26. Cognitive behavior therapy (CBT)
27. Hypnosis
28. Being admitted to a psychiatric ward of a hospital
29. Undergoing electroconvulsive therapy
30. Having an occasional alcoholic drink to relax
31. Going on a special diet or avoiding certain foods

32. Consulting a website that gives information about the problem
33. Consulting an expert using email or the web
34. Consulting a book giving information about the problem
35. Receiving information from a health educator

Q3

What is most likely to result for John/Mary given that he/she received the sort of professional help that you thought most appropriate. Select one option:

A. Full recovery with no further problems
B. Full recovery, but problems will probably recur
C. Partial recovery
D. Partial recovery, but problems will probably recur
E. No improvement
F. Get worse

Q4

What is most likely to result for John/Mary given that he/she does not receive any professional help? Select one option:

A. Full recovery with no further problems
B. Full recovery, but problems will probably recur
C. Partial recovery
D. Partial recovery, but problems will probably recur
E. No improvement
F. Get worse
